# Editorial: Mechanical circulatory support therapy for biventricular failure

**DOI:** 10.3389/fcvm.2024.1421550

**Published:** 2024-05-22

**Authors:** Jamshid H. Karimov, Kiyotaka Fukamachi, Toru Masuzawa

**Affiliations:** ^1^ Department of Biomedical Engineering, Lerner Research Institute, Cleveland Clinic, Cleveland, OH, United States; ^2^ Department of Biomedical Engineering, Cleveland Clinic Lerner College of Medicine of Case Western Reserve University, Cleveland, OH, United States; ^3^ Kaufman Center for Heart Failure, Heart, Vascular, and Thoracic Institute, Cleveland Clinic, Cleveland, OH, United States; ^4^ Ibaraki University, Mito, Japan

**Keywords:** heart failure, biventricular failure, mechanical circulatory support, BVAD, device therapies

**Editorial on the Research Topic** Mechanical circulatory support therapy for biventricular failure

Heart failure (HF) is a major public-health concern and one of the most frequent reasons for hospitalization. A primary contributor to cardiovascular mortality that affects approximately 64 million people worldwide, the prevalence of HF is rising rapidly, even in developing nations (1). HF typically occurs on the left side of the heart, but cardiac dysfunction can actually expand and impact the right side, causing biventricular failure (2, 3). Clinical experience with pharmacological therapy or a resynchronization device has been very limited. While the latter can provide symptomatic and survival benefits, many patients nevertheless develop progressive symptoms, refractory to further medical therapy; thus, ultimately requiring some sort of mechanical circulatory support (MCS) device (4–6). The current research's topics primary aim has been to propose and discuss the management strategies for biventricular failure patients with MCS devices, such as: the left ventricular assist device (LVAD); the biventricular assist device (BVAD); and, the total artificial heart (TAH). The articles in this collections ranged from advanced-imaging protocols, to studies surrounding the pathophysiocial controbutors for cariomyopathy; and, from hemodyamic-assessment protocols to ultimatcontinous-flow MCS devices.

In their recent publication, Zhang et al., reported their state-of-the-art investigative work, which seeks to establish cardiovascular magnetic resonance reference values of left and right ventricular morphology and function, based on a large sample of healthy Chinese adults (*n* = 550). All subjects included were stratified by gender (i.e., men/women), and age (in units of decades). The measurements of biventricular end-diastolic, end-systolic, and stroke volumes, ejection fraction, and end-diastolic left ventricular wall thickness and mass were obtained. Thorough analysis suggested that biventricular structure and function were significantly associated with age and sex. In terms of medical and device therapy optimization, the study could potentially be used as a “reference standard” for the diagnosis, risk stratification, and prognosis evaluation of cardiovascular disease in clinical research and practice in Chinese populations.

Another study, Grupper et al., described the temporal changes in hemodynamic parameters, both before and after LVAD implantation, among patients—with/without elevated pulmonary vascular resistance (PVR)—which is ultimately associated with worse prognosis in HF patients. The HF patients who received continuous-flow LVAD (HeartMate II and HeartMate 3) and underwent right heart catheterization with PVR reversibility study before and after LVAD surgery. Patients were subsequently divided into three subgroups (i.e., normal, reversible, and non-reversible PVR). The hemodynamic parameters improved after LVAD implantation, regardless of baseline PVR and reversibility, and enabled heart transplantation in patients who were previously ineligible, due to non-reversible, elevated PVR. The non-reversible PVR was associated with significantly reduced baseline cardiac output, when compared to all other study groups. These data suggest that the mechanism of PVR-improvement, post LVAD, is not only driven by a reduction in pulmonary pressure, but also by a significant increase in cardiac output. The research also emphasized the importance of a thorough hemodynamic assessment across the HF population(s), and suggested considerations of potential LVAD candidates may expand in the future.

Kakuda et al., retrospectively analyzed patients who had undergone heart transplantation (HTx) at the University of Tokyo Hospital, Japan. Kakuda and a team of researchers compared both hemodynamics and clinical events, after HTx, in patients stratified by the severity of residual PVR, after LVAD implantation, for bridge to transplantation. The LVAD therapy included multiple continuous-flow mechanical support systems, approved for clinical use. This study demonstrated that patients with residual high-PVR under LVAD, exhibited an increase of right and left atrial pressure in the chronic phase, after HTx. The residual high-PVR under LVAD implantation corresponded to an increase of diuretic use in the chronic phase, after HTx.

Ono reported a series of unique cases (*n* = 4) of arrhythmogenic right ventricular cardiomyopathy (ARVC), an inherited cardiomyocyte disease, characterized by intractable ventricular arrhythmia. Some patients were observed to manifest both right ventricular dysfunction and HF symptoms. Fatal ventricular arrhythmia has been the primary cause of death in ARVC patients; the pathophysiology of HF, seen in an ARVC patient who is a candidate for HTx, represents an advanced stage of biventricular failure. A durable ventricular assist device, or TAH, may be an option to bridge to HTx, but there is presently a lack of consensus on device type and optimal support. Ultimately, the author reported long-term support cases of biventricular failure successfully bridged to HTx, using continuous-flow LVAD.

Kuroda et al., (Cleveland Clinic), discusses biventricular failure with post left ventricular failure pathogenesis, based on the classification of support duration. The MCS with BVAD and TAH options remain challenging, as the overall treatment-strategy of BVAD and TAH therapies are largely dependent upon the support duration. This Editorial presents articles that contributed to Research Topic which address the optimal types of support, suggestions for duration—in both acute and chronic biventricular HF scenarios—as well as MCS in advanced-stage congenital heart disease that may require biventricular support.

Another Cleveland Clinic work (Karimov et al.) reports on various device-based options for biventricular HF support, using two unique devices currently in development: a universal ventricular assist device (UVAD), which will be able to assist the left, right, or both ventricles, and a continuous-flow TAH (CFTAH), which will be a replacement option for the failing heart. In these efforts, the *in vitro* hemodynamic performances of two UVADs were compared to a CFTAH, acting as a BVAD, and which had been evaluated for the first time. Comprehensive bench assessments of two different BVAD setups demonstrated self-regulation and exceptional pump performance, for both (single- and dual-device) -biventricular HF support scenarios. For treating moderate and severe biventricular HF, dual-device and single-device supports both functioned well, with respect to atrial pressure regulation and cardiac output; significantly, the dual-UVAD setup yielded a better atrial pressure balance in all testing conditions.

These works suggest an exciting future featuring unique, multimodal, and multidisciplinary approaches in the modern treatment of patients with advanced HF. More research will be necessary to explore existing and, more importantly, innovative approaches that could further improve patient survival and hospital experiences.
